# Protection from chemotherapy- and antibiotic-mediated dysbiosis of the gut microbiota by a probiotic with digestive enzymes supplement

**DOI:** 10.18632/oncotarget.25778

**Published:** 2018-07-20

**Authors:** Thomas E. Ichim, Santosh Kesari, Kim Shafer

**Affiliations:** ^1^ Immune Advisors, LLC, San Diego, CA, USA; ^2^ John Wayne Cancer Institute at Providence Saint John's Health Center, Santa Monica, CA, USA; ^3^ Daily Body Restore, LLC, Wixom, MI, USA

**Keywords:** probiotic, digestive enzymes, microbiome, chemotherapy, cancer

## Abstract

There are numerous downstream consequences of marketed drugs like antineoplastic agents on the gut microbiome, an effect that is suggested to contribute to adverse event profiles and may also influence drug responses. In cancer, progress is needed toward modulation of the host microbiome to prevent off-target side effects of drugs such as gastrointestinal mucositis that result from gut dysbiosis. The objective of this study was evaluation of the bioactivity of a supplement consisting of capsules with a blend of 9 probiotic organisms of the genera *Lactobacillus* and *Bifidobacterium* plus 10 digestive enzymes, in protecting the human gastrointestinal tract from chemotherapy and an antibiotic. We used the Simulator of Human Intestinal Microbial Ecosystem (SHIME) model, an *in vitro* model of a stable colon microbiota, and introduced 5-fluorouracil (5-FU) and vancomycin as microbiome-disrupting drugs. The probiotic with digestive enzymes supplement, added in capsules at *in vivo* doses, improved fermentation activity in the colon reactors and accelerated the recovery of microbial populations following 5-FU/vancomycin treatment. The supplement restored the Bacteroidetes to Firmicutes ratios in the colon reactors, increased the diversity of microbiota, and induced the production of microbial metabolites that elicited anti-inflammatory cytokines in an *in vitro* model of intestinal inflammation. In the proximal colon, preventative administration of the supplement resulted in full recovery of the gut microbial community after cessation of 5-FU and vancomycin treatment. These results identify a probiotic with digestive enzymes formulation that protects against drug-induced gut dysbiosis, highlighting its potential utility as a component of routine cancer care.

## INTRODUCTION

The microbiome in the human gut is composed of 500–1000 distinct bacterial species and up to 10^14^ total bacteria that are responsible for not only intestinal health but also for regulation of the immune system. The human gut microbiome is dominated by four main phyla: Firmicutes, Bacteroidetes, Actinobacteria, and Proteobacteria [1]. Metagenomic analyses show that changes in the relative abundance of the two dominant bacterial divisions, the Bacteroidetes and the Firmicutes, represent functional indicators of the metabolic potential of the gut microbiota, as demonstrated in experiments comparing microbiota in obese vs. lean animals [2]. Specifically, experiments have shown that obese mice had a 50% reduction in Bacteroidetes and a proportional increase in Firmicutes as compared to lean mice [3]. It is also interesting that this intestinal microbiota alteration in ratios of these phyla in obese subjects is associated with local and systemic inflammation [4]. Indeed, in the gut microbiome, the Bacteroidetes to Firmicutes ratio is regarded to be of significant relevance in diverse conditions that are associated with inflammation, including not only obesity [5], but also in aging [6], irritable bowel syndrome [7], and colon cancer [8]. In these cases, the decreased ratios of Bacteroidetes to Firmicutes were modulated in response to treatment strategies for example, by weight loss [5]. In another example, probiotic feeding was used to ameliorate the gut dysbiosis caused by a high-fat diet in experimental animals, marked by recovery of Bacteroidetes and a proportionate reduction in Firmicutes [9]. Of particular relevance to the present subject matter, colorectal cancer is marked by gut dysbiosis including reduced microbial diversity in the feces of patients than controls [1], and in tumor tissue compared vs. with areas of the mucosa at least 10 cm away [10]. Among other differences, a taxonomy based analysis of the gut microbiome showed that Firmicutes was significantly more abundant in the gut microbiota of cancerous tissues than that of adjacent non-cancerous tissues [11]. The mechanisms behind how these specific compositional changes in microbiota contribute to disease are not fully understood. The short-chain fatty acids acetate and propionate are the main fermentation products of the *Bacteroidetes* phylum while butyrate is mainly produced by *Firmicutes*. However, despite the proportional increases in Firmicutes in colon cancer, butyrate concentrations, which functions in many contexts including mitigating inflammation, are decreased during colon carcinogenesis; therefore, it is possible that specific beneficial species within this phylum may be less predominant. Indeed, studies have shown that strains of *Lactobacillus* (Phylum: Firmicutes) and *Bifidobacterium* (Phylum: Actinobacteria) are diminished in colorectal cancer [1]. These common probiotic bacteria are lactate acid producers that are involved in anti-inflammatory responses, anti-cancer activity, and pathogen exclusion from gut colonization [1]. There is also a relationship between the lactic acid bacteria and the butyrate producers since the latter utilize lactate [12]. On this basis, there are numerous mechanisms by which gut microbiota dysbiosis leads to increased permeability, aberrant immune activation, and chronic inflammation, all of which can contribute to colorectal cancer initiation and progression [1]. This information raises the question as to whether specific probiotic formulations can be used to restore the microbial composition and the beneficial microbial metabolites in the gut

Intestinal microorganisms may determine the outcome of cancer treatments as well as being themselves affected by the treatments. Immunotherapeutic drugs, such as checkpoint inhibitor antibodies designed to unmask the cancer patient's immune system, rely on a degree of endogenous immunity for their effectiveness, which is shown in several recent papers to be heavily influenced by the intestinal microbiome. For example, gut dysbiosis, evaluated based on stool samples, predicted resistance to immunotherapeutic interventions in melanoma patients [13]. Low diversity of commensal microorganisms was also associated with immune suppression in cancer patients [14]. Also, in a study of the anti-cancer effects of anti-PD1 or anti-PD-L1 checkpoint inhibitor antibodies, *Bifidobacteria,* a common probiotic strain, were found to be abundant in the colons of experimental animals that exhibited effective immunity against melanoma [15]. These lines of evidence provide testimony to the impact that the healthy gut microbiome has for cancer patients’ clinical outcomes. Specific therapeutics are also directly toxic to the gut microbiome and compromise patients’ recovery, notably, chemotherapeutic agents that can cause several side effects with gastrointestinal (GI) mucositis being one of the most frequent. Broadly speaking, chemotherapeutic agents cause changes in the microbiome that compromise energy metabolism, cause inflammation, and underlie the adverse events and poor quality of life of patients undergoing treatment. An example that has been studied is 5-fluorouracil (5-FU), first line agent for the treatment of metastatic colorectal agent, and a causative agent of severe colonic mucositis indicated by weight loss, diarrhea, bloody stool, shortened colon, and infiltration of inflammatory cells [16]. 5-FU diminishes bacterial richness and diversity in the gut, leading to reduced overall abundance of important phyla involved in normal microbial metabolism [16]. In fact, causal relationships are established between 5-FU-induced perturbations of the gut microbiota, the preponderance of pro-inflammatory cytokines in the intestinal milieu, and adverse events experienced by patients [16]. In another report that is of particular relevance to the present study, fecal microbiome transplantation was used to reverse antibiotic (ampicillin) and 5-FU-induced gut dysbiosis in a mouse model. Specifically, restoring the intestinal microbial composition to a healthy state by fecal microbiome transplantation successfully restored microbial diversity and richness, increased the composition of species known to exhibit anti-inflammatory actions such as *Lactobacillus,* and a coordinate reduction in known pathogenic strains [17]. Other drugs that are used in the armamentarium for cancer patients have also been proven to cause gut dysbiosis. Oral vancomycin, an antibiotic, is the mainstay of therapy for severe infections produced by *Clostridium difficile*, the most prevalent cause of healthcare-associated infectious diarrhea in developed countries. In cancer patients, bacterial infections occur through diagnostic and therapeutic procedures, for example, due to placement of central venous catheters, and may have particularly serious consequences owing to the underlying immunosuppression. During vancomycin therapy, most intestinal microbiota genera are depleted [18]. The rate of recovery of the microbiota following cessation from vancomycin determines whether the individual is subsequently susceptible to intestinal colonization by pathogenic bacteria. Since the duration of gut dysbiosis is a critical factor in managing adverse events of the treatment, evaluation of putative therapies such as probiotics should include monitoring the microbiota dynamics.

On the basis of this evidence, it has been widely suggested that modulation of the gut microbiome may be a useful therapeutic approach for improving the toxic side effects of cancer treatments, thereby possibly altering the trajectory of cancer. While a range or individual probiotic microorganisms or other supplements have been widely touted as having the capacity to modulate the gut microbiome, the challenge in the field has been the lack of validation of specific products. This is of particular significance for indications where patients are immunocompromised and undergoing treatment with cytotoxic agents since it has even been suggested that probiotics could induce bacteremia in certain populations [19–25]. Therefore, the potential utility of probiotic-containing supplements for specific indications remains unleveraged as a preventative means for offsetting adverse events associated with cancer therapeutics and for improving patients’ outcomes.

To address the need for a probiotic formulation that protects against gut dysbiosis, this study evaluated a proprietary blend of 9 probiotic organisms of the genera *Lactobacillus* and *Bifidobacterium*, as well as 10 digestive enzymes, for its efficacy at protecting the gastrointestinal (GI) tract from the combined effects of 5-Fluorouracil (5-FU) and vancomycin. The probiotic with digestive enzymes supplement was evaluated using the Simulator of Human Intestinal Microbial Ecosystem (SHIME), a stable and reproducible *in vitro* system, to analyze its influence chemotherapy plus antibiotic-induced changes in microbial community activity and composition. The present experiments provided a very stringent test for the effects of this dietary supplement since the dysbiosis created in this system represented a “two-hit” system implementing two known microbiome-disrupting agents. We report that the probiotic with digestive enzymes supplement beneficially modulated the gut microbiome under healthy conditions, and, most significantly, improved recovery from 5-FU/vancomycin treatment when administered to the SHIME system. Improvements in microbial fermentation and attenuation of microbial community dysbiosis elicited by 5-FU/vancomycin were observed when the probiotic with digestive enzymes supplement was given in a curative context (i.e. beginning at the time of 5-FU/vancomycin treatment) but particularly when given in a preventative context (i.e. starting prior to 5-FU/vancomycin).

## RESULTS

### Stability of the SHIME system for analysis of the effects of a probiotic with digestive enzymes supplement

The SHIME *in vitro* model system was designed to create a stabilized microbiota community to allow for collection of samples from the different intestinal regions for analysis. Several microbial parameters were monitored throughout the SHIME experiment to assess the performance of the model and the basic changes in the microbial community composition and activity due to the probiotic with digestive enzymes supplement. After the first week during the stabilization period, reproducibility between each of the three SHIME units was determined in order to confirm initiation of preventive treatment in arm 3 of the SHIME. Reproducibility was confirmed by analysis of SCFA levels, which were 85.8% similar between the SHIME units. During the control period, stability and reproducibility of the other two SHIME units was determined. SCFA levels were stable within (on average 86.8% similar between consecutive time points in control period) and reproducible between the two SHIME units (on average 86.8% similar), clearly indicating stability and reproducibility of the microbial community in terms of activity and composition. On this basis, it was concluded that the effects that would be observed during the subsequent treatment period could be attributable to the probiotic with digestive enzymes supplement rather than due to variability in the system itself.

### Modulation of acid/base consumption by the probiotic with digestive enzymes supplement that is indicative of increased fermentation activity

**Figure 1:**
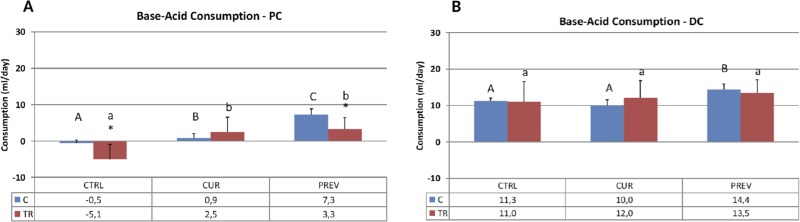
The pH in the SHIME system is maintained by pre-set pH controllers at 5.6–5.9 in the proximal colon (PC) and at 6.6 to 6.9 in the distal colon (DC) to ensure optimal environments for microbiota As the reactors acidify during changes in microbial activity, base is added. These results show acid and base consumption during control (**C**) and chemotherapy/antibiotic treatment (TR) periods for the control (CTRL), curative (CUR), and preventative (PREV) arms with the probiotic with digestive enzymes supplement. Results are shown for the reactors corresponding to the PC (**A**) and DC (**B**), and represent the average base/acid consumption over the entire control (*n* = 6 measurement) and treatment (*n* = 12) period. (^*^) represents statistically significant differences between C and TR (i.e. before and after 5-FU/vancomycin addition to the rea ctors, respectively). The different letters above the bars denote statistical comparisons between the indicated groups where uppercase or lowercase letters define distinct comparator groups; *p* < 0.05 was considered significant.

The consumption of acid and base reflects the overall microbial activity in the SHIME reactors representing the proximal and distal colon. Generally speaking, there are distinct bacterial populations that are native to the proximal and distal colon regions, reflecting the different requirements for digestion in each segment. Figure 1 depicts the average weekly acid/base consumption during the control and treatment periods (i.e. before and after 5-FU/vancomycin treatment). It should be noted that the treatment period consisted of one week of 5-FU/vancomycin followed by three weeks without these agents during which time recovery from 5-FU/vancomycin could be monitored and compared for the experimental treatment arms, as follows: 1) Control arm: receiving no supplement; 2) Preventative arm that had already been receiving the probiotic with digestive enzymes supplement all through the previous stabilization and control periods; and. 3; The curative arm that commenced supplementation with the probiotic with digestive enzymes supplement only at the start of 5-FU/vancomycin administration.

As per Figure 1, 5-FU/vancomycin treatment caused significant changes in reducing base consumption the proximal colon but not the distal colon. In the proximal colon, the addition of the probiotics with digestive enzymes in both the curative and preventative treatment arms significantly increased base consumption in the proximal colon, countering the effects of 5-FU/vancomycin. It should be noted that, *in vivo*, more bacterial fermentation activity also occurs in the proximal colon where substrate availability is higher [26]. In the distal colon, the probiotic with digestive enzymes supplement administered in the preventative arm also increased base consumption, however, the 5-FU/vancomycin itself did not significantly diminish this overall marker of metabolic activity in the distal colon reactors.

### Improvements in Short Chain Fatty Acid (SCFA) production by the probiotic with digestive enzymes supplement, revealing significant reversals of 5-FU/vancomycin-associated changes

The abundant SCFA, acetate, propionate and butyrate, are generated by fermentation of dietary fibers by gut microbiota. SCFA have a plethora of health-promoting effects through their interactions with metabolite-sensing G protein-coupled receptors on the gut epithelium and on immune cells [27]. In these experiments, we monitored the production of these three SCFA in the proximal and distal colon reactors, comparing pre- and post-5-FU/vancomycin treatments in the control (non-supplemented), curative, and preventative arms that were treated with the probiotic with digestive enzymes supplement.

**Figure 2:**
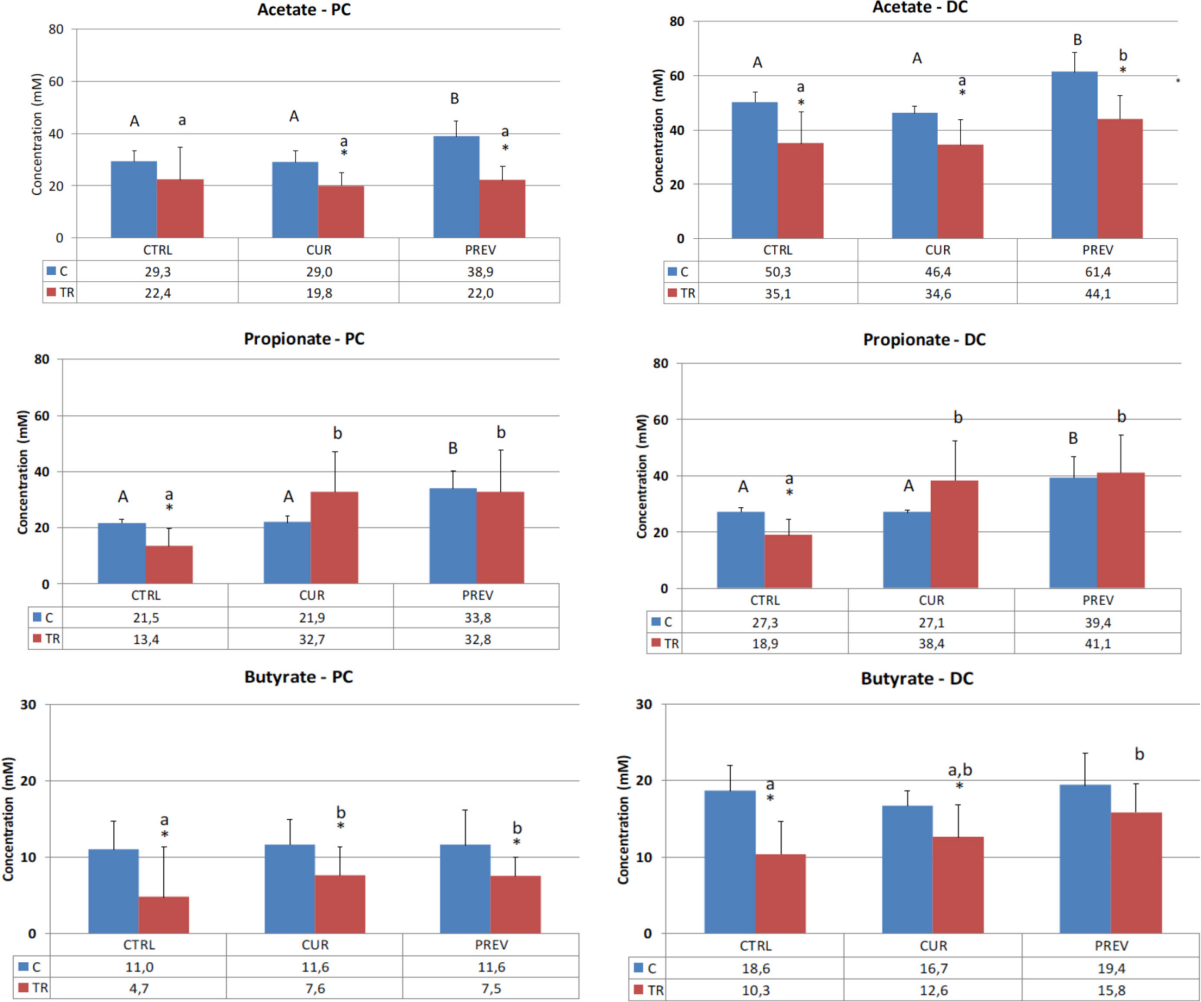
Effect of curative and preventative administration of a probiotic with digestive enzymes supplement on short chain fatty acid production in SHIME reactors corresponding to the proximal colon and distal colon Average acetate, propionate, and butyrate production, as indicated, over the control (C) (*n* = 6) and treatment (TR) (*n* = 12) periods for the control (CTRL), curative (CUR), and preventative (PREV) arms given the probiotic with digestive enzymes supplement is presented. (^*^) represents statistically significant difference relative to the preceding period. The different letters above the bars denote statistical comparisons between the indicated groups where uppercase or lowercase letters are used to delineate the distinct comparator groups, and *p* < 0.05 was interpreted as significant.

Figure 2 shows the results for acetate, which is produced by a range of gut microbes including *Bacteroides* and *Bifidobacteria*, and exerts anti-inflammatory effects [28]. Chemotherapy treatment resulted in a decrease in acetate levels in both the proximal and distal colon reactors. Although the recovery of acetate did not occur in the proximal colon, preventative treatment with the probiotic with digestive enzymes supplement increased acetate in both control and 5-FU/vancomycin treatment periods in the distal colon, suggesting that the pretreatment with the supplement offsets the adverse impact of the drugs on microbial metabolism.

In Figure 2, analysis of propionate concentrations in the SHIME is also provided, a product of a diverse group of metabolically active gut microbes, which exerts anti-inflammatory effects in the colon as well as systemically [28]. Once again, the expected reduction in propionate in the SHIME reactors was observed in the 5-FU/vancomycin treatment period vs. the control period in both the proximal and distal colon reactors. Here, both the curative and preventative treatment arms with the probiotic with digestive enzymes supplement resulted in recovery of propionate levels relative to the control arm.

Figure 2 also shows the results for butyrate production in the SHIME reactors, a primary product of *Clostridium* clusters IV and XIVa (phylum Firmicutes). *In vivo*, butyrate is largely metabolized by intestinal epithelial cells where it serves as an energy source as well as a homeostatic factor for normal colonic cell turnover and repair processes [28]. In the SHIME, the expected result was obtained whereby 5-FU/vancomycin treatment strongly decreased butyrate levels in both the proximal and distal colon reactors. Supplementation of the reactors with curative and preventative administration of the probiotic with digestive enzymes supplement resulted in improved butyrate production in the proximal and distal colon. Notably, the differences between the curative and preventative arms were not statistically different in either of the reactors.

### Changes in gut microbiota by the probiotic with digestive enzymes supplement, revealing an effect in countering 5-FU/vancomycin-induced gut dysbiosis

**Figure 3:**
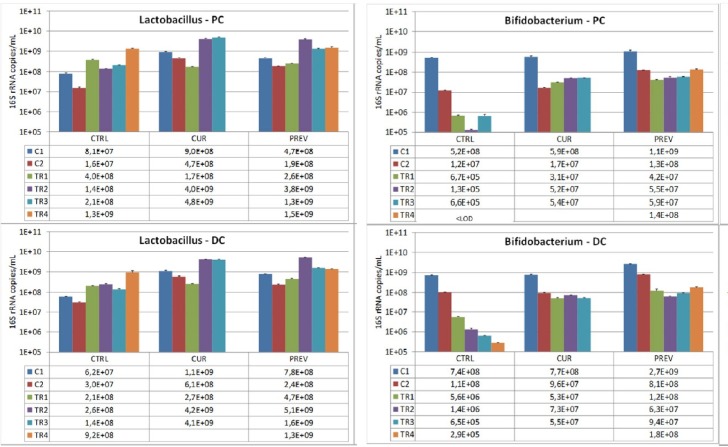
Analysis of modulation of the *Lactobacillu*s and *Bifidobacterium* composition by the probiotic with digestive enzymes supplement The effect of a curative (CUR) and preventive (PREV) administration of the probiotic with digestive enzymes supplement as compared to a control SHIME (CTRL) on luminal *Lactobacillus* (left panels) and *Bifidobacterium* (right panels) levels (16S rDNA copies/mL) in the proximal (PC; top panels) and distal colon (DC; bottom panels). The data are represented for the control weeks (C1, C2) and treatment weeks (TR1, TR2, TR3, and TR4. It should be noted that 5-FU/vancomycin was administered to the system in TR1 and discontinued in TR2–TR4. Preventative administration of the probiotic with digestive enzymes supplement was being administered throughout the control periods (C1 and C2), while curative supplementation of the probiotic with digestive enzymes formulation was initiated and maintained at TR1-TR4. (^*^) indicates statistically significant differences relative to the preceding period, while different letters indicate a statistical difference between different treatments; *p* < 0.05.

**Figure 4:**
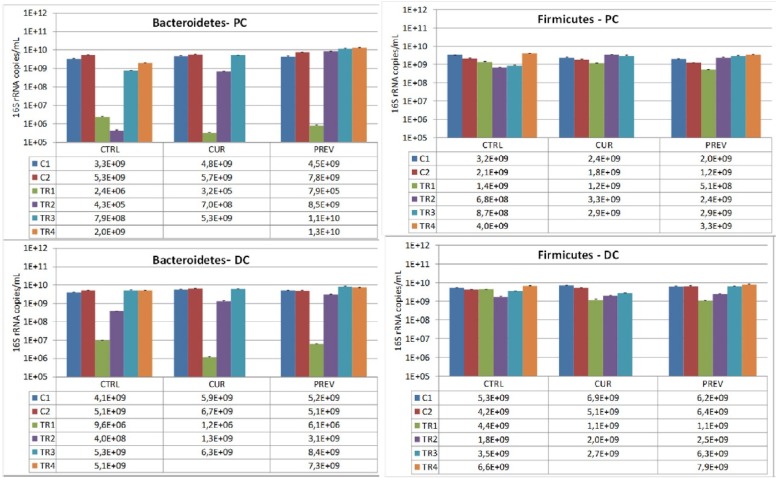
Changes in the dominant phyla of beneficial gut microbes, Bacteriodetes and Firmicutes, by the probiotic with digestive enzymes supplement The effect of a curative (CUR) and preventive (PREV) administration of the probiotic with digestive enzymes supplement as compared to a control SHIME (CTRL) on luminal Bacteroidetes, and Firmicutes levels (16S rDNA copies/mL) in the proximal (PC; top panels) and distal colon (DC; bottom panels). The data are represented for the control weeks (C1, C2) and treatment weeks (TR1, TR2, TR3, and TR4. It should be noted that 5-FU/vancomycin was administered to the system in TR1 and discontinued in TR2-TR4. Preventative administration of the probiotic with digestive enzymes supplement was being administered throughout the control periods (C1 and C2), while curative supplementation of the probiotic with digestive enzymes formulation was initiated and maintained at TR1-TR4. (^*^) indicates statistically significant differences relative to the preceding period, while different letters indicate a statistical difference between different treatments; *p* < 0.05.

To further evaluate the impact of the probiotic with digestive enzymes supplement, the next series of experiments utilized qPCR to identify changes the microbial composition in the SHIME reactors representing the proximal and distal colon. These analyses focused on looking at the rates of recovery of healthy microbiota following the administration of 5-FU/vancomycin during treatment week 1 (TR1; refer to Figures 3 and 4), where 5-FU/vancomycin was discontinued during the recovery weeks spanning TR2-TR4 (Figures 3 and 4). Comparisons were also conducted relative to the control period of the SHIME where no 5-FU/vancomycin had been added but the preventative arm was already receiving the probiotic with digestive enzymes supplement, allowing its influence on a healthy microbiome to be evaluated.

Specific strains of *Lactobacillus* and *Bifidobacterium* strains exert beneficial influences on maintenance of integrity of intestinal tissue in inflammatory circumstances and actions against toxicity of chemotherapeutic agents including intestinal mucositis [29–33]. In addition to being permanent genera of the human intestinal microbiota, these strains are highly enriched in the probiotic plus digestive enzymes supplement. We evaluated the SHIME reactors for the presence of these lactate-producing bacteria, the *Lactobacillus* and *Bifidobacterium* strains. In Figure 3, data are shown for control and 5-FU/vancomycin treatments among the different experimental arms that received no supplement or either curative or preventative administration of the probiotic with digestive enzymes supplement. First, as we expected, the results showed that 5-FU/vancomycin reduced the populations of these bacteria in the SHIME. For *Lactobacillus*, after the 5-FU/vancomycin treatment period during TR1 (Figure 3), *Lactobacillus* levels recovered after administration of the probiotic with digestive enzymes supplement, but also for the negative control in both colon regions. Significantly however, treatment with the supplement resulted in a faster recovery as compared to the negative control that lacked the supplement (Figure 3). For *Bifidobacteria*, after cessation of 5-FU/vancomycin, the levels of these microbes dropped drastically for the negative control in both colon regions, but especially in the PC where *Bifidobacteria* levels dropped below detection limit at the end of the treatment period (Figure 3). Administration of the probiotic with digestive enzymes supplement resulted in a slight recovery of *Bifidobacteria* levels, with no differences observed between the curative and the preventive arms of supplementation. It should be noted that, during the control periods (C1 and C2) prior to 5-FU/vancomycin treatment, overall *Lactobacilli* and *Bifidobacteria* did not increase in the PC or the DC.

We also assessed the composition of Bacteroidetes and Firmicutes, the predominant phyla in the healthy human gut, to determine how they are influenced by the probiotic with digestive enzymes supplement in the SHIME. The results showed that Bacteroidetes levels were particularly depleted during the 5-FU/vancomycin treatment period. Significantly, we observed that administration of the probiotic with digestive enzymes supplement also resulted in faster recovery of Bacteroidetes after 5-FU/vancomycin (after TR1) as compared to the control arm, an effect that was the greatest for the preventative arm of supplementation over the curative arm (Figure 4). Remarkably, the Bacteroidetes levels were restored to control (pre-5-FU/vancomycin) levels during treatment weeks 3 and 4. This finding supports the capacity of the supplement to hasten the recovery of the gut microbiota following 5-FU/vancomycin treatment with the lowest levels observed in the colon reactors on treatment week 2 (TR2; Figure 4). Analysis of Firmicutes showed a more modest depletion in response to 5-FU/vancomycin that was evident at treatment week 1 (Figure 4), however, all three arms (control, preventative, and curative) recovered in the colon reactors after the treatment was discontinued. Interestingly, as can be appreciated in the proximal colon reactors, there was a faster recovery of Firmicutes in the preventative and curative arms vs. the control arm. This recovery was noted during TR2 and TR3 where the probiotic with digestive enzymes supplement promoted the rapid return to the Firmicutes levels in the control period. Overall, these results show that the probiotic and digestive enzymes supplement restores the Bacteroidetes to Firmicutes ratios following 5-FU/vancomycin.

### Increased diversity of the microbial community is promoted by administration of the probiotic with digestive enzymes supplement

16S-targeted Illumina sequencing was used whereby amplified 16S rRNA marker gene sequences are clustered into taxonomic units of bacteria. When the data had been processed at the phylum and family levels, and the Simpson diversity index was calculated. The lowest possible value of the index is 1, representing a community consisting of only one Operational Taxonomic Unit (OTU). The highest possible value is the total number of OTUs, and the higher the index, the larger the diversity and the larger the evenness. Table 1 shows the Simpson Diversity Index results to evaluate the impact of the probiotic with digestive enzymes supplement on diversity changes in the microbiota in the SHIME that are caused by 5-FU and vancomycin. In the distal colon, the supplement given preventatively had the most significant impact on increasing the microbial diversity. In the proximal colon, the highest diversity was observed in the negative control (i.e. no supplement) following recovery from 5-FU and vancomycin (TR4). Curative treatment with the supplement also increased the microbial diversity in both the proximal and distal colons.

**Table 1:** Changes in the diversity of microbiota resulting from curative or preventative administration of the probiotic with digestive enzymes supplement

	Control		Curative		Preventative	
	Control	Treatment	Control	Treatment	Control	Treatment
Proximal Colon	3.0	7.6	2.7	5.4	2.2	2.6
Distal Colon	7.5	7.5	7.5	9.2	11.5	11.2

Reciprocal Simpson Diversity Index in the lumen of the proximal and distal colon of the SHIME upon a curative (CUR) and preventive (PREV) administration of the probiotic with digestive enzymes supplement was compared to a control SHIME (CTRL) at the end of the control [control week 2 (C)] and treatment [treatment week 4 (TR4)] periods. The intensity of the shading indicates the absolute diversity index, normalized for each of the environments (i.e. within each row).

**Figure 5:**
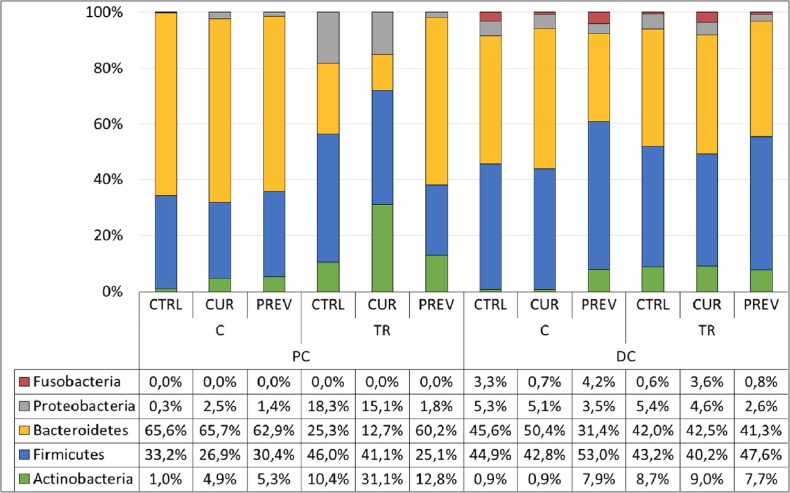
Addition of the probiotic with digestive enzymes supplement to the SHIME modulates the microbial communities at the phylum level Abundance (%) of the dominant phyla in the lumen of the proximal (PC) and distal colon (DC) of the SHIME upon a curative (CUR) and preventive (PREV) administration of the probiotic with digestive enzymes supplement as compared to control (CTRL) the end of the control week 2 (C) and treatment week 4 (TR) period.

Lastly, 16S-targeted Illumina sequencing was used to evaluate the differences in microbial compositions at the phylum level in the proximal and distal colons caused by the probiotic with digestive enzymes supplement. In Figure 5, the results show that the supplement did indeed cause changes in the microbial communities. The preventative treatment prior to 5-FU/vancomycin treatment did not cause any major changes at the phylum level in the proximal colon. However, in the distal colon, preventative supplementation resulted in increases in abundance of Actinobacteria and reductions in Bacteroidetes and Proteobacteria.

Following 5-FU/vancomycin treatment, the microbiome in the distal colon did not differ at the phylum level for the control, curative or preventative arms (Figure 5). In contrast, in the proximal colon, only preventive administration of the probiotic with digestive enzymes supplement resulted in full recovery of the gut microbiota after cessation of 5-FU/vancomycin treatment, mainly increasing the abundance of Bacteroidetes at the expense of Proteobacteria as compared to the other experimental arms. Collectively, these findings support the most significant changes in beneficial microbial communities as occurring in response to preventative administration with the probiotic with digestive enzymes supplement. However, it is plausible that a longer duration of curative treatment with the supplement would afford similar results as the preventative treatment.

### Impact of the probiotic with digestive enzymes supplement on cytokines in an *in vitro* model of intestinal inflammation

We next asked how the probiotic with digestive enzymes supplement alters the composition of bacterial-derived metabolites having the potential to impact immune function as a means of offsetting the negative effects of 5-FU/vancomycin. To this end, we leveraged an *in vitro* model system that has been described as an ‘inflammatory bowel disease-like’ model that uses Caco-2 (intestinal epithelial like cells) and THP-1 macrophages [34]. Caco-2 cells originate from a human colon adenocarcinoma cell line that can differentiate into mature, enterocyte-like cells that are characterized by the formation of villi, presence of tight junctions, and expression of apical brush border enzymes, thereby recapitulating the colon [35]. THP-1 cells, derived from acute leukemia, differentiate into macrophages upon culture with phorbol 12-myristate 13-acetate (PMA), and can then be activated toward a highly pro-inflammatory phenotype upon treatment with lipopolysaccharide (LPS). In the setup used here, Caco-2 cells were placed on top of PMA-treated THP-1 cells, on the apical and basolateral sides of culture chambers, respectively. Colonic suspensions collected from the SHIME reactors, treated with the same regimens of 5-FU/vancomycin and probiotic with digestive enzymes supplement as in the previous experiments, were added to the apical side of the culture chambers containing Caco-2 cells. After 24 h of the apical pre-treatment of the Caco-2/THP-1 co-cultures with the SHIME samples, the basolateral supernatant was discarded and the THP cells were treated with LPS to provide inflammatory signals. Subsequently, after 6 hours of stimulation, cytokines were measured from the basolateral side of the chamber containing the THP-1 cells, which will have been affected indirectly by signals from the Caco-2 cells or directly by the transport of metabolites and molecules. In this manner, the interactions between cells of the gut and the immune system can be recapitulated *in vitro.* These experiments allow examination of the influence of the microbial fermentations-derived products following preventive and curative administration of the probiotic with digestive enzymes supplement in the face of 5-FU/vancomycin treatment.

**Figure 6:**
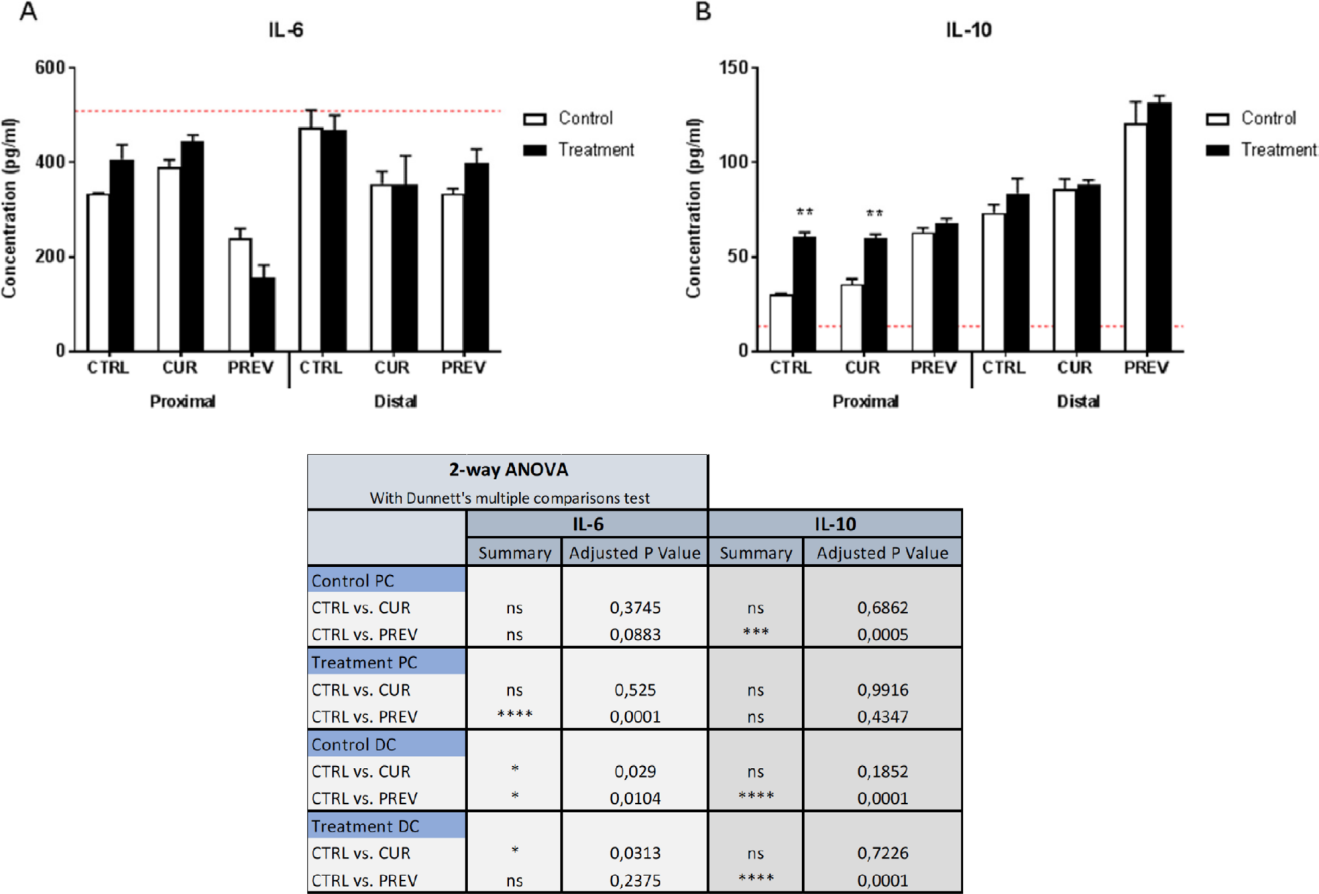
Modulation of cytokine profiles in an *in vitro* model of intestinal inflammation by metabolites from SHIME reactors treated with the probiotic with digestive enzymes supplement Samples of SHIME suspensions were taken from reactors with curative (CUR) and preventive (PREV) administration of the probiotic with digestive enzymes supplement as compared to a control SHIME (CTRL; no supplement) from the control period (baseline microbial community prior to 5-FU/vancomycin) and treatment period with 5-FU/vancomycin. Cytokine levels of IL-6 (A) and IL-10 (B) were measured 6 h after LPS treatment on the basolateral side of the Caco2/THP-1 co-cultures after pre-treatment of the apical side for 24 h with the SHIME samples. The red dotted line corresponds to the experimental control consisting of LPS. Data are plotted as the mean ± SEM. The chart (lower panel) outlines the corresponding statistical analyses performed using two-way ANOVA with Dunnett's multiple comparisons test. Significance is depicted where (^*^). (^*^), (^**^), (^***^) and (^****^) represent *p* < 0.05, *p* < 0.01, *p* < 0.001 and *p* < 0.0001, respectively. Ns = non-specific; PC: proximal colon; DC: distal colon.

The results of the Caco-2/THP-1 co-culture experiments are provided in Figure 6. Measurement of IL-6 was performed as known pro-inflammatory signature of LPS-stimulated macrophages, and also of colonic mucosal cells [36, 37]. The results showed that preventative treatment with the probiotic with digestive enzymes supplement generated microbial metabolites in the proximal colon reactors that decreased IL-6 concentrations in the co-culture model as compared to the control reactors that were not supplemented but were treated with 5-FU/vancomycin. Thus, LPS-induced inflammation was modulated by fermentation products derived from reactors to which the supplement was administered prior to treatment with 5-FU/vancomycin. From the distal colon reactors, the SHIME suspensions taken from the control period, during which baseline microbial activities were monitored, and the treatment period with 5-FU/vancomycin did not differ with respect to the concentrations of IL-6 in the co-culture. Secondly, we also monitored IL-10 production induced in the Caco-2/THP-1 cultures as a representative anti-inflammatory cytokine produced by macrophages during LPS stimulation (Figure 6). One point of interest was that that the metabolites from SHIME reactors following treatment with 5-FU/vancomycin induced greater IL-10 production than those from the control (baseline) period in the PC. However, administration of the probiotic with digestive enzymes supplement prior to 5-FU/vancomycin (i.e the control period) generated metabolites in the PC with the highest IL-10 inducing capabilities in the co-cultures. In the DC, the results showed statistically significant increases in IL-10 were generated in co-cultures given SHIME suspensions from the preventative arm from both the control period and 5-FU/vancomycin treatment period. Collectively, the reductions in IL-6 and increases in IL-10 observed in the Caco-2/THP-1 model of intestinal inflammation containing SHIME suspensions suggest that the probiotics with digestive enzymes supplement affects the gut microbiome toward controlling inflammation.

## DISCUSSION

The objective of this study was to evaluate the potential benefits of a proprietary probiotic with digestive enzymes supplement in preventing the gut dysbiosis caused a chemotherapy treatment and an antibiotic in a pre-clinical analysis of bioactivity. The test product contains a blend of 9 probiotic organisms of the *Lactobacillus* and *Bifidobacterium* genera, as well as 10 digestive enzymes contained in capsules that were administered in an *in vitro* system to recapitulate the gut microbiome using doses of the product that would be administered clinically. Using the SHIME model platform, where the gut microbiota can be cultured under representative conditions of the different intestinal regions, we successfully demonstrated that the probiotic with digestive enzymes supplement was capable of modulating the effects of 5-FU/vancomycin when administered in a preventative context. We also noticed positive changes in the microbial activity and composition using the probiotic with digestive enzymes supplement added to the SHIME beginning at the same time as 5-FU/vancomycin (i.e. the curative arm), including the production of metabolites that induced anti-inflammatory cytokine production in the Caco-2/THP-1 model. Based on studies that are ongoing, we believe that a longer duration of curative supplementation than was performed in the SHIME would have achieved at least a similar magnitude of beneficial effects as the preventative arm.

The present study bridges the large gap that exists between the extensive microbiome research taking place and the lack of validation for specific products that are on the market. To this point, one DNA-based analysis of bifidobacterial species in commercial probiotic products demonstrated that only one of sixteen tested products matched the bifidobacterial label claim, and pill-to-pill and lot-to-lot variation was observed [38]. The authors of this study also pointed out that misidentified commercial products, in addition to the lack of comparison between strains or species, is a barrier to the ability of clinicians to make informed decisions about what to prescribe. Another point of discussion is that there is a great deal of variability between products in terms of how many viable, functional probiotic bacteria can be delivered to the gut; therefore, testing of effectiveness of supplements using advanced *in vitro* analysis is advisable [39]. Another understudied area, which, to our knowledge, has not been previously examined in pre-clinical studies, is a supplement that combines probiotic microorganisms with digestive enzymes. While digestive enzymes generally serve to improve nutrient absorption in the gut, they also are expected to have a complementary or synergistic role with the probiotic microbes. One study reported that pancreatic enzyme supplementation to experimental animals modified the intestinal microbiota, allowing increased colonization of *Lactobacillus* strains in addition to improving nutritional status [40]. In a study related to microbial activities in the genital tract, amylase, a glycogen-degrading enzyme, was found to allow for the growth of certain *Lactobacillus* isolates in the glycogen breakdown products that cannot grow in absence of the enzyme [41]. These studies support a beneficial impact of combining probiotics with digestive enzymes to improve microbial metabolism in the gut.

Cancer patients undergoing chemotherapy, radiation therapy or other drug treatments experience a plethora of adverse events related to their treatments including nausea, vomiting, diarrhea, and loss of appetite, leading to a lower dietary intake, weight loss, and susceptibility to infections. These symptoms can be directly linked to the disturbed intestinal microbiome. During gut dysbiosis, parameters such as water volume, ion concentrations, osmotic pressure and pH are often abnormal, leading to growth of bacteria that further contribute to homeostatic imbalances and mediate aggressive inflammation [42]. This inflammation damages the intestinal mucosa, reducing the overall microbial diversity and allowing pathogenic species to thrive in the gut. Indeed, pyrosequencing studies have demonstrated that probiotics can afford a 5-fold decrease in the abundance of members of the genus *Fusobacterium* [43], potential pathogens that are enriched in the mucosal flora of colorectal cancer patients [44]. By introducing missing microbial components, probiotics are believed to directly antagonize enteric pathogens, modulate innate or adaptive immunity, and strengthen mucosal barrier function to alleviate gastrointestinal symptoms [45]. Cancer cachexia, a metabolic disorder characterized by anorexia and muscle wasting, represents another unmet medical need. Interestingly, in an animal model of leukemia that is characterized by cachexia, administration of an oral probiotic to restore *Lactobacillus* species reduced the expression of markers of atrophy in the muscles as well as inflammatory cytokines including IL-6 [46]. Similarly, administration of prebiotics in a cachexia model led to modulation of the gut microbiome, including increases in beneficial strains such as *Bifidobacterium,* which coincided with metabolic shifts in the periphery and a delay in tumor-induced cachexia [47]. Conservation of the gut microbiome may therefore improve not only tumor- and treatment-associated changes the gut itself but also systemic metabolic disturbances.

A separate and related issue is the role of the gut microbiome in tumor initiation since there is abundant evidence that chronic infection and the ensuing inflammation are contributing factors to tumor initiation [48]. SCFA promotes and maintains colonic epithelial health through maintaining barrier function, suppressing tumorigenesis by regulating DNA methylation and diminishing oxidative stress, and inhibiting inflammation (Sun) It is plausible that dietary changes or probiotic interventions may reduce the risk of colorectal cancer, however, the potential impact of microbiome modulating-strategies on tumorigenesis itself is not clear, and awaits investigation for the probiotics with digestive enzymes supplement that was the focus of the current study.

The limited evidence confirming the safety and benefits of these products in human clinical studies has led to a recommendation for cancer patients, who are immunocompromised, to take only moderate doses of dietary supplements or none at all [49]. Isolated case reports have asserted that overt bacteremia can result from the use of probiotics by immunocompromised patients. Several cases of *Lactobacillus* and *Bifidobacterium* bacteremia [19–25] as well as cases of sepsis associated with *Saccharomyces cerevisiae* [50, 51], a probiotic strain used the treatment and prevention of *Clostridium difficile*-associated diarrhea, have been reported to be associated with probiotic intake by ICU patients and individuals with immunodeficiency disorders. The amassed results from 17 studies revealed 5 case reports of probiotic-associated bacteremia or fungaemia based on blood culture tests. Another analysis [52] concluded that “there remain insufficient studies to assess the true effect of probiotics in people with cancer. Meta-analysis suggests probiotics may be beneficial but further studies are still required. Improved reporting of outcomes and adverse events in clinical trials are required to improve accuracy and confidence of conclusions drawn in future updates.” With respect to efficacy, another a recent meta-analysis carried out to analyze data related to the efficacy and safety of probiotics in people with cancer, the results compiled for 11 studies showed that probiotics may reduce the severity and frequency of diarrhea in patients with cancer and may reduce the requirement for anti-diarrheal medication, however, the analysis showed that firm conclusions could not be drawn with the available information [53]. We would argue, and it is our approach, that research and clinical studies need to be performed in a product-specific manner to evaluate for a positive impact on the microbiome and for efficacy for a particular indication. In sum, using a dynamic model of the gut ecosystem, this report validates a probiotic with digestive enzymes supplement as being beneficial for countering microbial imbalances and maladaptation in the gastrointestinal tract. These results provide an impetus for clinical studies evaluating the curative and preventative effects of this supplement against chemotherapy-associated adverse events that stem from gut dysbiosis.

## MATERIALS AND METHODS

### Probiotic with digestive enzymes supplement

The probiotic with digestive enzymes supplement used herein comprises capsules is manufactured using proprietary methods and contains a blends of probiotics (116.20 mg total weight); specifically, *Bifidobacterium infantis*, *Bifidobacterium bifidum, Lactobacillus acidophilus, Lactobacillus salivarius, Lactobacillus plantarum, Lactobacillus rhamnosus, Bifidobacterium longum, Lactobacillus casei, Lactobacillus paracasei,* and digestive enzymes (272.65 mg total weight); specifically, amylase, glucoamylase, lipase, bromelain, maltase, lactase, hemicellulose, xylanase, papain, and invertase.

The capsules used herein are the same formulation that would be administered *in vivo*. The product was tested at an *in vitro* dose of 3 capsules/day before chemotherapy/antibiotic treatments, and 4 capsules/day during and after chemotherapy/antibiotic treatment (to be described below). This corresponds to an *in vivo* dosage of 6 capsules/day and 8 capsules/day before and after chemotherapy/antibiotic treatment, respectively.

### Simulator of human intestinal microbial ecosystem (SHIME) setup

ProDigest (Gent, Belgium) conducted the SHIME experiments and analysis. Briefly, the SHIME system consists of a series of double-jacketed vessels, simulating the digestive compartments that are initially inoculated with a fecal sample from a healthy adult donor using methods described previously [54].

The typical reactor setup consists of a succession of five reactors simulating the different parts of the human gastrointestinal tract. The first two reactors simulate different steps in food uptake and digestion, with peristaltic pumps adding a defined amount of SHIME feed (140 mL 3×/day) and pancreatic and bile liquid (60 mL 3×/day), respectively to the stomach (V1) and small intestine (V2) compartment and emptying the respective reactors after specified intervals [54]. The last three compartments simulate the large intestine. These reactors are continuously stirred, and they have a constant volume and pH control. Retention time and pH of the different vessels are chosen in order to resemble *in vivo* conditions in the different parts of the colon. Upon inoculation with fecal microbiota, these reactors simulate the ascending (V3), transverse (V4) and descending (V5) colon.

The present experiments employed an adapted SHIME setup to accommodate the following three treatment arms (vs. two arms that are typically compared using this system):

Control Arm: Chemotherapy and antibiotics; no supplement given.

Curative Arm: Chemotherapy and antibiotics; probiotic with digestive enzymes supplement added at the same time as the other agents.

Preventative Arm: Chemotherapy and antibiotics; probiotic with digestive enzymes supplement added prior to the other agents.

Accordingly, a TripleSHIME set-up was utilized wherein the colon reactors were limited to two instead of three to account for the additional test conditions required; specifically, reactors corresponding to PC-DC units (proximal and descending colon) instead of AC-TC-DC units (ascending, transverse, and descending colon).

### Stages of the SHIME experiment

The stages of the experiment are described below and also summarized in Table 2.

**Table 2:** Overview of treatment stages with 5-fluorouracil and vancomycin in the SHIME

Week 1	Week 2	Week 3	Week 4	Week 5	Week 6	Week 7	Week 8
Stabilization	Stabilization	Control	Control	Treatment 5-FU/vancomycin	Recovery from treatment	Recovery from treatment	Recovery from treatment

### Stabilization period (Weeks 1 and 2)

After inoculation of the colon reactors with an appropriate fecal sample, a two-week stabilization period allowed the microbial community to differentiate in the different reactors depending on the local environmental conditions. During this period, the basic nutritional matrix was provided to the SHIME to support diversity of the gut microbiota originally present in the fecal inoculum. The third arm of the SHIME setup (preventive arm; PREV), already received 3 capsules/day during the stabilization period (corresponding to an *in vivo* dose of 6 capsules/day).

### Control period (Weeks 3 and 4)

During this two-week reference period, the standard SHIME nutrient matrix was further dosed to the model. Analysis of samples in this period allows to determine the baseline microbial community composition and activity in the different reactors, which will be used as a reference for evaluating the treatment effects. The third arm of the SHIME setup (PREV), already received 3 capsules/day during the control period (~ *in vivo* dose of 6 capsules/day).

### Treatment period (Weeks 5–8; 5-FU plus vancomycin during Week 5)

During this four-week period, the SHIME reactor was operated under nominal conditions, but each arm was treated as follows. During the first week, all arms received chemotherapy (10 μM of 5-fluorouracil (5-FU)) and vancomycin treatment (62.5 mg/L of vancomycin). During the subsequent weeks, it was considered a “recovery” period from the effects of 5-FU and vancomycin. Arm 1 of the SHIME did not receive any additional treatment (i.e. control arm; CTRL). Arm 2 of the SHIME received the probiotic with digestive enzymes supplement (4 capsules/day ~ *in vivo* dose of 8 capsules/day) at the start of the chemotherapy administration (i.e. curative arm; CUR), whereas addition of the supplement for arm 3 of the SHIME was continued (at 4 capsules/day ~ *in vivo* dose of 8 capsules/day) (PREV).

### Analysis of the activity and composition of microbiota in the SHIME

The following microbial parameters were monitored throughout the entire SHIME experiment to evaluate the performance of the model and/or to allow monitoring of basic changes in the microbial community composition and activity due to the probiotic with digestive enzymes supplement.

### Microbial community activity

The concentrations of SFCA; specifically, acetic acid, propionic acid and butyric acid, were analyzed as by-products of microbial metabolism. Each of these parameters was measured three times/week. Briefly, SCFA were extracted from samples with diethyl ether after the addition of 2-methylhexanoic acid as an internal standard. Extracts were analyzed using gas chromatography as described previously [55]. The concentrations of lactate, the precursor of SCFA, were also monitored using a d-lactate/l-lactate kit (R-Biopharm, Mannheim, Germany), according to the manufacturer's protocols.

### Microbial community composition

As part of the SHIME experiments, the following groups were quantified via quantitative PCR (qPCR; once/week): Bacteroidetes phylum, Firmicutes phylum, *Lactobacillus* spp. and *Bifidobacterium* spp. as previously reported [56].

### 16S-targeted Illumina sequencing

16S ribosomal RNA (rRNA) sequencing was used to analyze samples from the SHIME reactors to identify and compare the microorganisms using similar methods as published elsewhere [57]. The Illumina sequencing method was used to amplify microbial sequences until a saturation level was reached. Information on a broad spectrum of OTUs was obtained (>100 different of the most dominant OTUs), however, the results were presented as proportional values versus the total amount of sequences within each sample, thus providing semi-quantitative results. The methodology used primers that span 2 hypervariable regions (V3–V4) of the 16S rDNA. Using a paired sequencing approach, sequencing of 2×250 bp resulted in 424 bp amplicons.

To provide an ecological interpretation of these data, the Simpson reciprocal index was calculated as a measure of diversity and evenness of the microbiota as described previously [58]. An increase in the Simpson reciprocal index reflects a diversity increase, with 1 being the lowest possible number, and the number of bacterial species/OTUs present in the sample being the maximal number. The index will approach the maximal value when the OTU distribution is more even. The higher the index, the larger the diversity and the larger the evenness.

### *In vitro* analyses of immune markers

#### Caco-2 cells

The co-culture experiment was performed as previously described [34] Briefly, Caco-2 cells (HTB-37; American Type Culture Collection) were seeded in 24-well semi-permeable inserts (0.4 μm pore size) at a density of 1 × 10^5^ cells/insert. Caco-2 monolayers were cultured for 14 days, with three medium changes/week, until a functional cell monolayer was obtained. Cells were maintained in Dulbecco's Modified Eagle Medium (DMEM) containing 25 mM glucose and 4 mM glutamine and supplemented with 10 mM HEPES and 20% (v/v) heat-inactivated (HI) fetal bovine serum (FBS).

#### THP-1 cells

THP1 cells (InvivoGen) were maintained in Roswell Park Memorial Institute (RPMI) 1640 medium containing 11 mM glucose and 2 mM glutamine, supplemented with 10 mM HEPES, 1 mM sodium pyruvate and 10% (v/v) HI-FBS. THP1 cells were seeded in 24-well plates at a density of 5 × 10^5^ cells/well and treated with 100 ng/mL of PMA for 48 hours (h). PMA induces the differentiation of the cells into macrophage-like cells.

#### Caco-2/THP-1 co-cultures

To mimic the interface between host immune cells and the fermentation products of the gut microbiome, *in vitro* experiments were conducted based on previous studies by Satsu and colleagues [59]. In this setup, the colonic suspensions collected from the SHIME are brought in contact with the apical side of the co-cultures (i.e. Caco-2 cells). The effects observed on the basolateral chamber where the THP-1 cells reside are mediated indirectly by signals produced by the Caco-2 cells and/or by the transport of micro- and macro-molecules.

Briefly, the apical compartment containing Caco-2 cells was filled with sterile-filtered (0.22 μm) colonic SHIME suspensions (diluted 1:5 (v/v) in Caco-2 complete medium). Cells were also treated apically with 12 mM Sodium butyrate (NaB) (Sigma-Aldrich) as a positive control in experiments establishing the system. The basolateral compartment containing THP1 cells was filled with Caco-2 complete medium. Cells were also exposed to Caco-2 complete medium in both chambers in control experiments. Cells were treated for 24 h, at which time the basolateral supernatant was discarded and THP-1 cells were stimulated with Caco-2 complete medium containing 500 ng/mL of ultrapure LPS (*Escherichia coli* K12, InvivoGen). Cells were also stimulated at the basolateral side with LPS in combination with 1 μM hydrocortisone (HC) (Sigma-Aldrich) and medium without LPS (LPS-) in control experiments. Cultures were incubated at 37 degrees Celcius in a humidified atmosphere of air/CO_2_ (95:5, v/v). After 6 h of LPS stimulation, the basolateral supernatants were collected for cytokine measurement (human IL-6 and IL-10) by Luminex. multiplex (Affymetrix-eBioscience) according to the manufacturers’ instructions. All treatments were done in triplicate.

### Statistics

To evaluate the difference between the treatment sample and its respective control sample within each SHIME, a two-way ANOVA with Sidak's multiple comparisons test was performed. These results are presented on the graphs. To evaluate the difference between the SHIMES (CUR and PREV) compared to the CTRL SHIME within each colon compartment for both the control and the treatment phase, a two-way ANOVA with Dunnett's multiple comparisons test was performed. These results are presented in the figures where (^*^) represents *p* < 0.05. All statistics were performed using GraphPad Prism version 7.02 for Windows (GraphPad Software, San Diego, CA, USA).

For the analysis of the cytokines IL-6 and IL-10, statistical significance was calculated between the different SHIMES (CUR and PREV) compared to the CTRL SHIME using two-way ANOVA with Dunnett's multiple comparisons test was performed. Results were considered significant if *p* < 0.05.
